# Retraction: SDR9C7 Promotes Lymph Node Metastases in Patients with Esophageal Squamous Cell Carcinoma

**DOI:** 10.1371/journal.pone.0227461

**Published:** 2020-01-06

**Authors:** The PLOS ONE Editors

Following publication of this article [1], concerns were raised about regions of overlap between some image panels in Figs 3A, 4E and 4G. Specifically:

- There is a region of overlap between the image panels for the EC9706-N Migration Assay and the EC9706-N Invasion Assay in Fig 3A.
- There is a region of overlap between the EC109-P Invasion Assay panel in Fig 3A and the EC9706-P Migration Assay panel in Fig 4G.
- There is a region of overlap between the EC109-P Migration Assay panel in Fig 3A and the Con-EC9706-P Invasion Assay panel in Fig 4G.
- There is a region of overlap between the image panels for the EC109-P Migration Assay and the EC9706-P Migration Assay in Fig 3A.
- There is a region of overlap between the EC9706-P Migration Assay panel in Fig 3A and the EC109-P migration Assay panel in Fig 4E.
- There is a region of overlap between the EC109-P Invasion Assay panel in Fig 4E and the EC109-P Invasion Assay panel in Fig 3A.
- There is a region of overlap between the EC109-P Invasion Assay panel in Fig 4E and the EC9706-P migration Assay panel in Fig 4G.
- There is a region of overlap between the Si-EC109-P Migration Assay panel in Fig 4E and the Si-EC9706-P migration Assay panel in Fig 4G.
- There is a region of overlap between the Con-EC9706-P Invasion Assay panel in Fig 4G and the Con-EC109-P Migration Assay panel in Fig 4E.

The corresponding author has stated that there were unintentional errors in image selection and placement, and that this did not affect the cell counts and statistical analyses or the final conclusions of the study. The authors provided the underlying dataset for the invasion, migration and MTT assays shown in Figs 3 and 4, as well as the underlying image files for Figs 3A, 4E, and 4G, and replicate images captured during the original experiments. Replacement images were provided for the incorrect panels; however, the corresponding author indicated that records were not kept relating to the cell types, conditions, and experimental design associated with the image files.

Additional concerns were raised regarding the reporting of the microarray experiment. The corresponding author indicated that experiments and data analysis related to Fig 1 were performed by an external company, which was not stated in the published article. The complete microarray data set was not initially made available. The authors have deposited the microarray data at GEO (accession number GSE118493). The corresponding author has acknowledged there were some errors in the reporting of the microarray results. Specifically:

- The following up-regulated transcripts were omitted from Table 2: A_23_P60990 C2orf54 and A_24_P175187 SAMD9.
- The following down-regulated transcripts were omitted from Table 3: A_23_P62901 BTG2, A_23_P203120 CADM1, A_24_P342388 DMD, A_23_P129925 SLFN11.
- There is an error in the volcano plot in Fig 1b, caused by input of incorrect data during construction of the plot. Up-regulated and down-regulated genes were not separated prior to log transformation of FCAbsolute, resulting in a volcano plot with superposition of dots representing both up- and down-regulated genes. The corresponding author has stated that this mistake was not caused by and had no effects on the data generated by the microarray analysis. A revised volcano plot was provided.

The concerns regarding the reporting of the microarray experiment and the availability of the microarray data were addressed during the editorial follow up. However, due to the incomplete records, it was not possible to resolve concerns regarding the accuracy of the original published images in Figs 3A, 4E and 4G or the replacement images provided. In light of concerns about the preparation of Figs 3A, 4E and 4G, and the reliability of the reported results, the *PLOS ONE* Editors retract this article.

The authors did not confirm agreement or disagreement with the retraction decision.

## References

[1] Tang S, Gao L, Bi Q, Xu G, Wang S, Zhao G, et al (2013) SDR9C7 Promotes Lymph Node Metastases in Patients with Esophageal Squamous Cell Carcinoma. PLoS ONE 8(1): e52184 10.1371/journal.pone.0052184 23341893 PMC3544840

